# Phylogenetic Analyses of Trichodinids (Ciliophora, Oligohymenophora) Inferred from 18S rRNA Gene Sequence Data

**DOI:** 10.1007/s00284-012-0274-5

**Published:** 2012-11-30

**Authors:** Fa-Hui Tang, Yuan-Jun Zhao, Alan Warren

**Affiliations:** 1 The Key Laboratory of Animal Biology of Chongqing, Chongqing Normal University, Chongqing, 400047 People’s Republic of China; 2 Department of Zoology, Natural History Museum, Cromwell Road, London, SW7 5BD UK

## Abstract

Partial 18S rRNA gene sequences of the three trichodinids, namely *Trichodina modesta* Lom, 1970, *Trichodina paraheterodentata* Tang and Zhao 2012. and *Trichodinella epizootica* (Raabe 1950) Šrámek-Hušek, 1953, were acquired and used to construct phylogenetic trees. The results revealed that *Trichodinella epizootica* clustered with *Trichodinella* sp.; *Trichodina paraheterodentata* Tang and Zhao 2012 was sister to the clade composed of *Trichodina heterodentata* Duncan, 1977 and *Trichodina* *nobilis* Chen, 1963; *Trichodina modesta* Lom, 1970 clustered with *Trichodina reticulata* Hirschman and Partsch, 1955. The branching order of species within the Mobilia clade was closely correlated with GC content. Furthermore, blade morphology was also found to be the primary morphological character in determining the phylogenetic relationships among members of the genus *Trichodina*. The present findings suggest that the genus *Trichodina* is paraphyletic when species of *Trichodinella* are included in the analyses.

## Introduction

Members of the family Trichodinidae are best known as ectoparasites of fishes. About 300 species of trichodinids have been described from fishes, mostly from freshwater environments [35]. In China, the trichodinid ciliates of freshwater fishes have received considerable attention in recent years [12, 13, 22–31, 36, 39–42]. Hitherto, most studies have focused on their morphology following silver impregnation. However, morphological characters have proved inadequate to reconstruct evolutionary history as many are unique to the sub-class Mobilia so their weighting is difficult to determine and some, such as the presence or absence of central granules in the adhesive disc, lack a consensus as to their systematic importance. Molecular data are increasingly used for studying phylogenetic relationships among ciliates. However, there have been relatively few such studies of mobilians prompting calls for more sequence data for taxa within this group [8, 9, 33, 37].

In this article, we sequenced the small subunit rRNA (18S rRNA) gene of three trichodinids, namely *Trichodina modesta* Lom, 1970; *Trichodina paraheterodentata* Tang and Zhao, 2012 and *Trichodinella epizootica* (Raabe 1950) Šrámek-Hušek 1953, in order to analyse their molecular phylogeny. The main aims of this work are to increase knowledge and understanding of the diversity and phylogeny of trichodinids. The importance of denticle blade morphology, central granules and GC content in the phylogeny of trichodinids are also discussed.

## Materials and Methods

### Collection and Identification (Fig. 1)

Specimens of host fishes, *Siniperca chuatsi* (ca. 1 year old, 15–35 cm in length), *Misgurnus anguillicaudatus* (ca. 1 year old, 10–26 cm in length) and *Carassius auratus* (ca. 1 year old, 8–25 cm in length) were collected from the Jialing River in the urban zone of Chongqing, China between February 2005 and April 2009. Each host was necropsied and examined under a binocular dissecting microscope (NIKON SMZ1500) at 400× in order to detect trichodinids. Fresh gill or skin smears containing trichodinids were prepared and impregnated using the dry silver method of Klein [11]. The nuclear apparatus was revealed using the methyl green-pyronin stain [6]. Observations, counts and measurements on impregnated specimens were performed using a compound microscope (NIKON E600, Nikon Instrument Inc., Shanghai, China) at a magnification of 1,000 × . Systematics follows Lynn (2008) [17] and Zhan et al. (2009) [37]. Terminology is mainly according to Corliss (1979) [4].

**Fig. 1:**
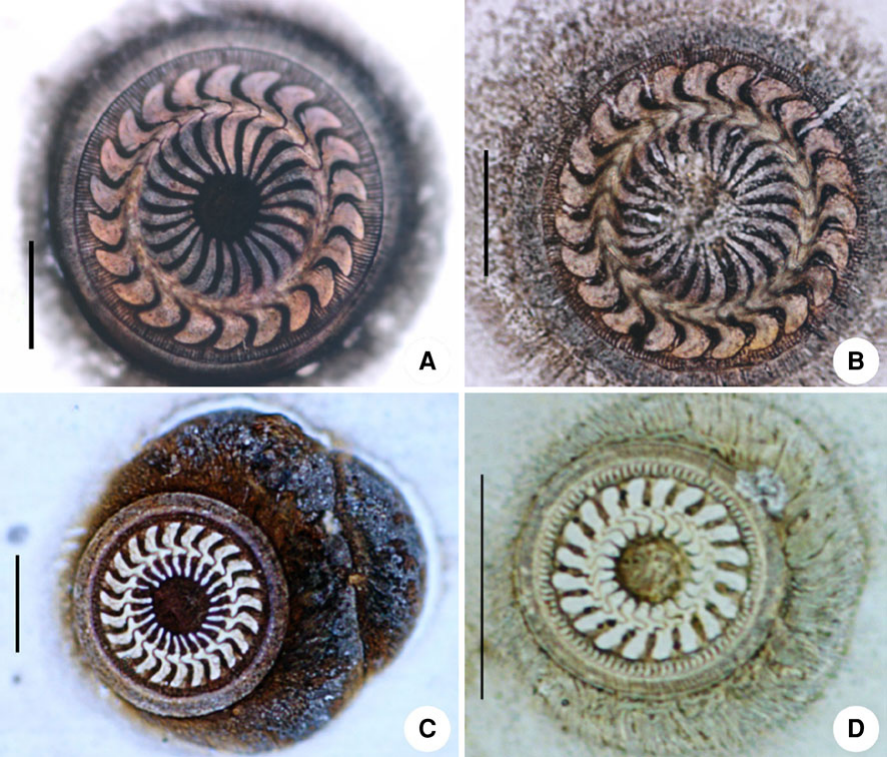
Photomicrographs of silver-impregnated adhesive discs of three trichodinids. A–B *Trichodina paraheterodentata* Tang and Zhao 2012 (from *Siniperca chuatsi*); C *Trichodina modesta* Lom, 1970 (from *Misgurnus anguillicaudatus*); D *Trichodinella epizootica* (Raabe,1950) Šrámek-Hušek 1953 (from *Carassius auratus*). (*Scale bar* 20 μm)

### DNA Extraction, Amplification, Cloning, and Sequencing

For each trichodinid species, at least 4 or 5 individuals were harvested, washed several times in a PCR tube and centrifuged at 6000–7500×*g*. DNA was extracted using REDExtract-N-AmpTM Tissue PCR Kit (Sigma, St. Louis, USA) following the manufacturer’s instructions.

The 18S rRNA genes of *T. paraheterodentata* and *T. epizootica* were amplified by the polymerase chain reaction (PCR) with the universal eukaryotic primers, forward primer 5′-AAC CTG GTT GAT CCT GCC AGT-3′, reverse primer 5′-TGA TCC TTC TGC AGG TTC ACC TAC-3′ [18]. Temperature cycling was five cycles of denaturation for 1 min at 94 °C, primer annealing for 2 min at 56 °C, and extension for 2 min at 72 °C, followed by 35 cycles in the same manner, but with the annealing temperature increased to 62 °C, and a final extended elongation step at 72 °C for 10 min. The 18S rRNA gene of *T. modesta*, was amplified with the primer pair MX5-MX3, forward primer 5′-CTG CGG ACG GCT CAGTAA ATC AGT-3′ and reverse primer 5′-CCA GGA CAT CTT AGG GCA TCA CAGA-3′ [1]. The cycling parameters were as follows: 5 min initial denaturation at 94 °C; then 35 cycles of 1 min at 94 °C, 1 min at 56 °C, and 2 min at 72 °C, followed by an extended elongation step at 72 °C for 10 min. Purified PCR products were inserted into a pMD18-T vector (TaKaRa) and selected clones were sequenced in an ABI Prism 377 DNA Sequencer (Applied Biosystems Inc., Foster City, California).

### Phylogenetic Analyses

The nucleotide sequences used for the present analyses are available from GenBank databases (for accession numbers see Table 1). A total of 24 complete or partial 18S rRNA gene sequences, including those of our three newly sequenced trichodinid species, were used to construct the phylogenetic trees. The hypotrich *Euplotes minuta* was the outgroup taxon. All sequences were first aligned using Clustal X 1.81 [32] and further modified manually using BioEdit 5.0.6 [10] with consideration of the secondary structures. Maximum likelihood (ML) and Bayesian Inference analyses were employed for tree construction. The ML tree was constructed in PAUP*4.0b10 [21]. Bootstrap confidence values were calculated with a heuristic search using simple sequence addition and 100 replicates. Bayesian analyses were conducted in MrBayes 3.1.2 [19] under a GTR model with 10^6^ generations, tree sampling every 100 generations, with a burn-in of 10000 trees to generate a posterior probability distribution using Markov chain Monte Carlo (MCMC) methods.

**Table 1:** GenBank accession numbers and sources of the 18S rRNA gene sequences of 24 ciliate species used in this study

Species selected for phylogenetic trees	Accession number	Authors (year)
*Euplotes minuta*	AY361908.1	Giuseppe et al. (unpublished)
*Epistylis wenrichi*	AF335515.1	Miao et al. (2001)
*Epistylis urceolata*	AF335516.1	Miao et al. (2001)
*Epistylis chrysemydis*	AF335514.1	Miao et al. (2001)
*Epistylis galea*	AF401527.1	Miao et al. (2004)
*Vorticella campanula*	DQ662849.1	Miao et al. (2001)
*Vorticella microstoma*	DQ868347.1	Clamp and Williams (2006)
*Vorticella convallaria*	DQ868348.1	Clamp and Williams (2006)
*Vorticella fusca*	DQ190468.1	Li and Song (Unpublished)
*Zoothamnium duplicatum*	DQ662851.1	Li and Song (2008)
*Zoothamnium nii*	DQ662852.1	Li and Song (2008)
*Zoothamnium pluma*	DQ662854.1	Li and Song (2008)
*Zoothamnium alternans*	DQ662855.1	Li and Song (2008)
*Trichodina reticulata*	AY741784.1	Gong et al. (2006)
*Trichodina heterodentata*	AY788099.1	Gong et al. (2006)
*Trichodina nobilis*	AY102172.1	Zhu et al. (2006)
*Trichodina ruditapicis*	FJ499385.1	Zhan et al. (2009)
*Trichodina sinonovaculae*	FJ499386.1	Zhan et al. (2009)
*Trichodina meretricis*	FJ499387.1	Zhan et al. (2009)
*Urceolaria urechi*	FJ499388.1	Zhan et al. (2009)
*Trichodina paraheterodentata*	GU906244	Present work
*Trichodina modesta*	GU906245	Present work
*Trichodinella epizootica*	GU906246	Present work
*Trichodinella* sp.	AY102176.1	Gong et al. (2006)

## Results

### GC content Analyses (Fig. 2, Table 2)

The GC contents of 18S rDNA for the 11 mobilian species used in the phylogenetic analyses are listed in Table 2. Five trichodinids have a GC content more than 50 %, namely *T. paraheterodentata*, *T. heterodentata*, *T. nobilis, Trichodinella* sp. and *T. epizootica.* Trichodinids with GC content between 48 and 50 % include the three marine species, *Trichodina ruditapicis*, *T. sinonovaculae* and *T. meretricis.* Two trichodinids have GC content between 46 and 48 %, i.e. *T. modesta* and *T. reticulata. Urceolaria urechi* has the lowest GC content, between 44 and 46 %.

**Fig. 2:**
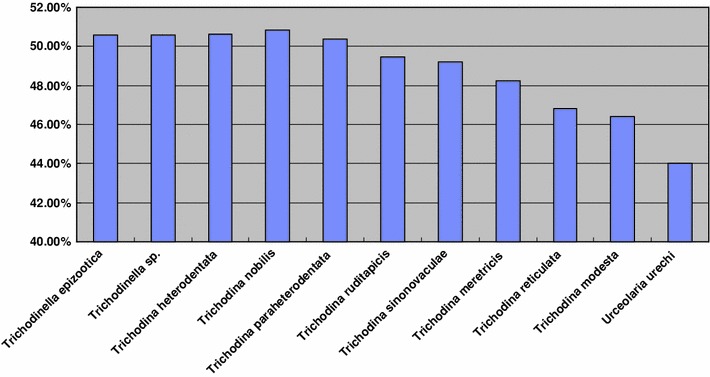
Bar chart of GC contents for 11 Mobilia species

**Table 2:** Comparison of GC contents and blade morphology for 11 Mobilia species

Species selected for phylogenetic trees	GC content	GC scope	Blade morphology
*Trichodina heterodentata*	50.65 %	≥50 %	Sickle-shaped (arc-shaped)
*Trichodina nobilis*	50.82 %	≥50 %	Fan-shaped (arc-shaped)
*Trichodina paraheterodentata*	50.38 %	≥50 %	Sickle-shaped (arc-shaped)
*Trichodinella epizootica*	50.56 %	≥50 %	Long strip-shaped
*Trichodinella* sp.	50.56 %	≥50 %	Long strip-shaped
*Trichodina meretricis*	48.23 %	48–50 %	Irregular quadrangle
*Trichodina ruditapicis*	49.47 %	48–50 %	Irregular quadrangle
*Trichodina sinonovaculae*	49.20 %	48–50 %	Irregular quadrangle
*Trichodina modesta*	46.43 %	46–48 %	Regular quadrangle
*Trichodina reticulata*	46.83 %	46–48 %	Regular quadrangle
*Urceolaria urechi*	44.04 %	44–46 %	Lamellar

**Fig. 3:**
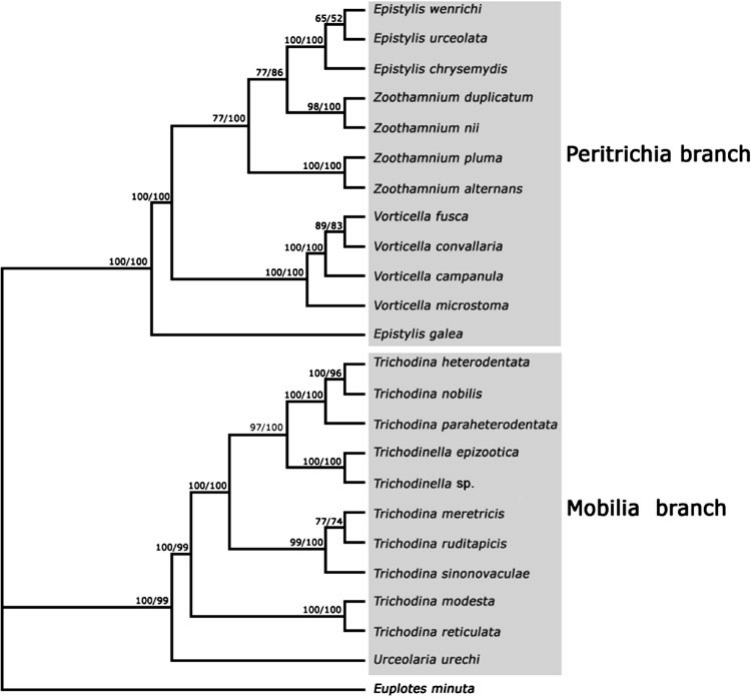
The phylogenetic tree of selected species of oligohymenophorean sub-classes Peritrichia *s. str*. and Mobilia inferred from small subunit rDNA sequences using maximum likelihood (ML) by Paup.4.10 and Bayesian analysis with the model of ‘‘GTR+1+G’’. The numbers at the nodes represent the bootstrap percentages from 1,000 replicates for ML and the posterior probabilities from 1,000,000 generations for Bayesian analysis, respectively

In the 18S rRNA gene trees, the branching order of the different clades corresponded with the GC content of species within each clade. For example, trichodinids with a GC content more than 50 %, i.e. *T. paraheterodentata*, *T. heterodentata*, *T. nobilis, Trichodinella* sp. and *T. epizootica,* clustered together in the terminal clade. These were preceded by a clade comprising three species, i.e. *T. ruditapicis*, *T. sinonovaculae* and *T. meretricis,* all of which have a GC content between 48 and 50 %. The clade that branched first within the trichodinid clade comprises two species, viz. *T. modesta* and *T. reticulata,* both of which have a GC content between 46 and 48 %. *U. urechi,* which branched basally within the Mobilia, possesses the lowest GC content, between 44 and 46 %. (Fig. 3).

### Phylogenetic Analyses (Fig. 3)

The phylogenetic trees, based on maximum likelihood and Bayesian inference analyses, respectively, had very similar topologies therefore only one tree is presented here (Fig. 3). This reveals that (1) the 23 species of Peritrichia s. str. and Mobilia were divided into two distinctly separate branches; (2) the urceolariid clade, represented by *U. urechi,* branched basally within the Mobilia and was sister to the trichodinid assemblage; (3) the two *Trichodinella* species clustered together in a clade nested within the trichodinid assemblage which otherwise comprises only species of *Trichodina*, suggesting the latter is paraphyletic; (4) the two species of *Trichodina* with central granules in the adhesive disc, namely *T.* *modesta* and *T. reticulata,* clustered together in a basal position within the trichodinid assemblage; (5) those species from marine mollusc hosts, i.e. *Trichodina sinonovaculae*, *T. meretricis* and *T. ruditapicis,* clustered together in a clade nested within the remaining trichodinids, all of which were isolated from freshwater fish hosts; (6) *T. paraheterodentata* did not cluster with *T. heterodentata* in any of the analyses but rather was sister to the clade comprising *T. heterodentata* and *T.* *nobilis.*

## Discussion

In the 18S rRNA gene trees, the peritrichs s.l. were divided into two well-supported clades: the sessilid forms or Peritrichia s.str. and the mobilian forms, which are now recognised as the sub-class Mobilia [37]. This finding is consistent with previous phylogenetic analyses of peritrichs based on gene sequence data such as 18S rRNA and α-tubuline [8, 9, 33, 37]. Representatives of three genera of mobilians were included in the present analyses: *Urceolaria, Trichodinella* and *Trichodina. Urceolaria* branched separately from the rest of the Mobilia, which was expected since it belongs to the family Urceloariidae as opposed to all other taxa in the analyses which are members of the family Trichodinidae. By contrast, the *Trichodinella* clade nested within the *Trichodina* assemblage. *Trichodinella* is separated from *Trichodina* by two main morphological characters: the development of the adoral rows of cilia which turn 180°–270° around the peristome in the former (vs. 360°–540° in *Trichodina*) and the denticles which have short, stunted thorns and delicate blades (vs. denticles robust with well-developed thorns and blades in *Trichodina*). These differences have long been considered sufficient for generic separation [15, 16].

Hitherto, gene sequence datum was available for only one species of *Trichodenella*, viz. *Trichodinella.*sp., which was ever reported by the name of *T. myakkae* [9]. This species has been included in at least two previous studies of mobilian phylogeny based on 18S rRNA gene sequence and in both cases it was nested within a larger *Trichodina* assemblage [9, 33]. Consequently, this called into question the identity of the genus *Trichodinella* [33], and doubts about the identity of *T. myakkae* resulted in its exclusion from the analysis by Zhan et al. [37]. In this study, a second species of *Trichodinella*, viz. *T. epizootica* the identity of which was confirmed by careful morphological examination (Fig. 1), was sequenced for the first time. As expected, *T. epizootica* had a high level of similarity (97 %) with *Trichodinella* sp. and the two clustered together with maximum bootstrap support, suggesting that they are congeneric. The inclusion of a second species of *Trichodinella* made no difference to the placement of this genus in the gene tree.

One factor that has not previously been taken into account when considering phylogenetic relationships among mobilians is GC content. In this study, it was noted that the branching order of the various clades closely corresponded with the GC content of the constituent species, those with a lower GC content (e.g. *U. urechi*, GC content 44–46 %) branching first, with each successive clade having increasing GC content, those with the highest GC content (i.e. *T. paraheterodentata* Tang and Zhao 2012, *T. heterodentata*, *T. nobilis, Trichodonella* sp. and *T. epizootica*) branching last. The GC content is traditionally regarded as being characteristic of the genome of any given organism and, in the case of bacteria, has been used in taxonomy and classification [7, 20]. Furthermore, Du et al. [5] used the GC levels of genome-wide genes to determine the correlation between the GC content and evolutionary relationships. This is consistent with the findings of Zhang et al. [38] who also reported a close association between GC content and evolutionary relationships among lichens. The biological significance of GC content is not fully understood. For example, Cao et al. [3] unexpectedly discovered the function of lower GC content in editing exons and revealed a possible relationship between molecular characteristics of DNA, RNA and purifying selection. Clearly, the influence of GC content on trichodinid phylogeny needs further investigation. Thus, there remain four possible explanations for the placement of *Trichodinella* in the 18S rRNA gene tree (1) that *Trichodinella* and *Trichodina* should not be separated at the level of genus; (2) the genus *Trichodina* is paraphyletic; (3) the placement of *Trichodinella* is an artifact and its true phylogenetic position is not recovered in the present analysis due to using just a single gene, undersampling etc.; (4) the placement of *Trichodinella* reflects its GC content rather than its true phylogenetic position.

**Fig. 4:**
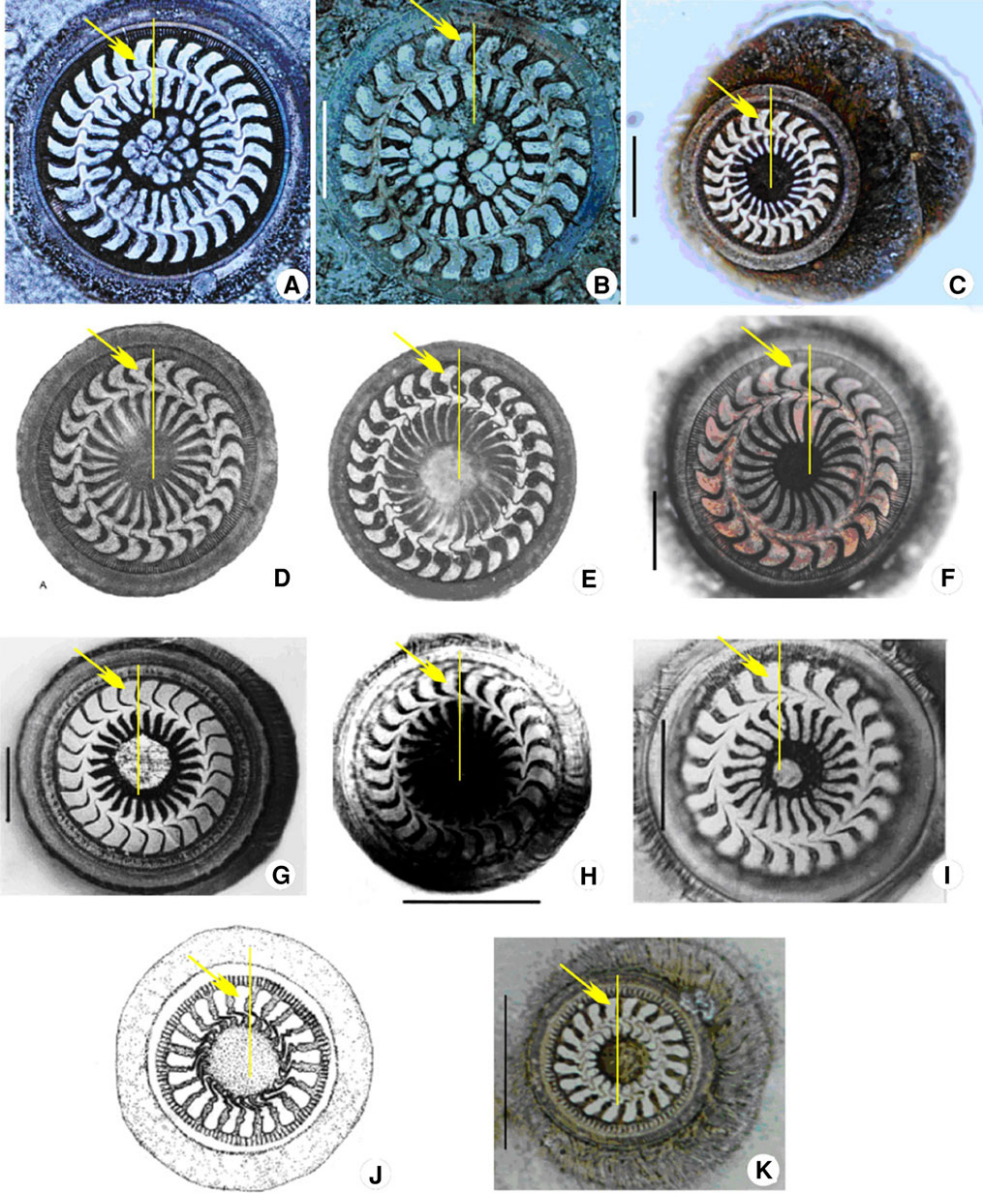
Denticle morphology of different trichodinids, arrows mark the blade of the denticle and the *yellow lines* indicate the *Y*-axis. A–B *Trichodina reticulata* (Tang and Zhao, 2010); C *Trichodina modesta* (present work); D *Trichodina heterodentata* (Gong et al. 2006); E *Trichodina nobilis* (Gong et al. 2006); F *Trichodina paraheterodentata* (Tang and Zhao 2012); G *Trichodina sinonovaculae* (Xu et al. 1999); H *Trichodina meretricis* (Xu et al. 1999); I *Trichodina ruditapicis* (Xu et al. 2000); J *Trichodinella* sp. (Gong et al. 2006); K *Trichodinella epizootica* (present work). (*Scale bar* 20 μm)

A morphological character that can be mapped onto the 18S rRNA gene tree with a high level of correlation is the shape of the denticle blade. Denticle morphology is an important character for the circumscription and identification of species and genera of trichodinids [2, 14, 34]. Therefore, it is not surprising that species with similar blade shapes tend to cluster together within the gene tree of the Mobilia (Figs. 4). For example, the three *Trichodina* species within the terminal clade, i.e. *T. paraheterodentata*, *T. heterodentata* and *T. nobilis*, all possess an arc-shaped blade; the two *Trichodinella* species, *Trichodina* sp. and *T. epizootica*, cluster together and both have long, strip-like blades; the three *Trichodina* species from marine mollusc hosts, *T. meretricis*, *T. ruditapicis* and *T. sinovaculae*, all have irregular quadrangular-shaped blades, and; the two species that branch basally within the trichodinid clade, *T. modesta* and *T. reticulata*, have a regular quadrangular-shaped blade (Fig. 4). These findings support the view that denticle blade shape is significant in the phylogeny of the Mobilia [37].

Another morphological character suggested as being of phylogenetic importance among mobilians is the presence or absence of central granules in the adhesive disc [9]. However, we could not find evidence to support this in this study, which is consistent with the findings of Zhan et al. [37]. For example *T. reticulata*, which possesses granules, is sister to *T. modesta*, which lacks granules. By contrast, there was evidence that phylogeny among mobilians may be influenced by the host and/or habitat with the three *Trichodina* species from marine mollusc hosts, *T. meretricis, T. ruditapicis* and *T. sinovaculae*, clustering together to the exclusion of the other species, all of which were isolated from freshwater fishes. This is consistent with Zhan et al. [37] who also remarked on the possible importance of co-evolution with the host in the phylogeny of mobilians.

Clearly gene sequence data are of growing importance in determining phylogenetic relationships among mobilians. However, undersampling remains a significant barrier to progress with sequence data being available for only 14 out of a possible ca. 300 mobilian species. Furthermore, with the notable exception of Gong et al. [8] who analysed the α-tubulin gene of 10 mobilian species, data are only available for the 18S rRNA gene. Thus, taxon sampling needs to be increased, and a wider range of genes analysed, before we can fully elucidate the phylogeny of the Mobilia.
